# Current Progress in Cross-Linked Peptide Self-Assemblies

**DOI:** 10.3390/ijms21207577

**Published:** 2020-10-14

**Authors:** Noriyuki Uchida, Takahiro Muraoka

**Affiliations:** Department of Applied Chemistry, Graduate School of Engineering, Tokyo University of Agriculture and Technology, 2-24-16 Naka-cho, Koganei, Tokyo 184-8588, Japan

**Keywords:** self-assembly, peptide, cross-link, hydrogel, tissue engineering

## Abstract

Peptide-based fibrous supramolecular assemblies represent an emerging class of biomaterials that can realize various bioactivities and structures. Recently, a variety of peptide fibers with attractive functions have been designed together with the discovery of many peptide-based self-assembly units. Cross-linking of the peptide fibers is a key strategy to improve the functions of these materials. The cross-linking of peptide fibers forming three-dimensional networks in a dispersion can lead to changes in physical and chemical properties. Hydrogelation is a typical change caused by cross-linking, which makes it applicable to biomaterials such as cell scaffold materials. Cross-linking methods, which have been conventionally developed using water-soluble covalent polymers, are also useful in supramolecular peptide fibers. In the case of peptide fibers, unique cross-linking strategies can be designed by taking advantage of the functions of amino acids. This review focuses on the current progress in the design of cross-linked peptide fibers and their applications.

## 1. Introduction

**Figure 1:**
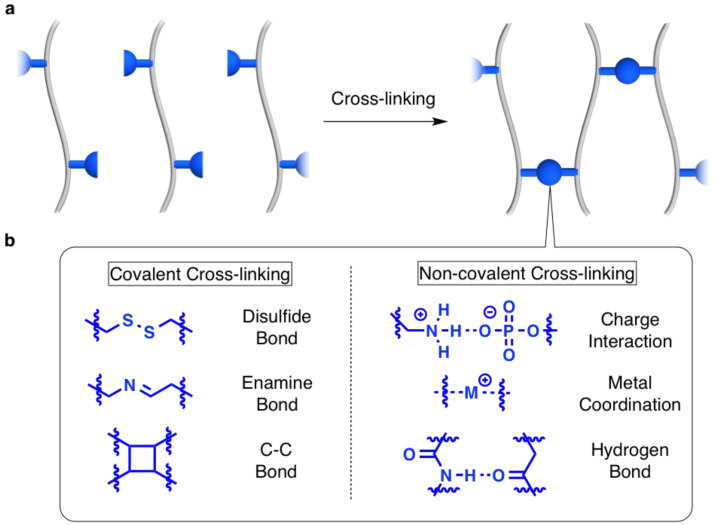
(**a**) Schematic illustration of strategy for cross-linked peptide fibers. (**b**) Chemical cross-linking based on covalent bonds such as disulfide bond, enamine bond, or C-C bond, and physical cross-linking based on non-covalent bonds such as charge interaction, metal coordination, or hydrogen bond are available depending on purposes.

There has been a lot of effort to create novel biomaterials applicable to tissue engineering and drug delivery systems. Among them, peptide-based fibrous supramolecular assemblies are one of the most attractive platforms to design biomaterials with various bioactivities and structures, and they have recently attracted particular attention. In pioneer works, amphiphilic peptides having hydrophobic alkyl chain at the termini of hydrophilic peptides have been designed, and their fundamental properties and applications to biomaterials have been investigated [1,2,3,4,5,6,7,8,9,10,11,12,13,14,15,16,17,18,19,20,21,22,23,24,25,26,27,28,29,30,31,32,33]. Currently, both amphiphilic peptides and other peptide-based self-assembly units have been found, enabling the design of peptide fibers with a variety of functions [34,35,36,37,38,39,40,41,42,43,44,45,46,47,48,49,50,51,52,53,54,55,56,57,58,59,60,61,62,63,64,65,66,67]. Cross-linking between peptide fibers is a powerful strategy to control their functions. The construction of peptide fiber networks in dispersions by cross-linking causes drastic changes in physical and chemical properties (Figure 1a). A representative example is hydrogelation, which converts peptide fiber dispersions into self-standing materials for biomaterial applications including cell culture scaffolds. Cross-linking methods, which have been extensively developed using covalent water-soluble polymers [68,69,70,71,72,73,74,75,76,77,78], are also effective in the cases of supramolecular peptide fibers. In particular, unique cross-linking methods can be employed by taking advantage of the functionalities of amino acid residues in peptide fibers [79,80,81,82,83,84,85,86,87,88,89,90]. Enzymes enable mild and specific reactions with substrates even in physiological conditions, which is advantageous to design biocompatible cross-linked peptide fibers. As a result of this, cross-linking using enzymes such as lysine oxidase (LOX) has also been significantly developed [20,54]. Generally, two types of cross-linking, chemical and physical cross-linking, can be chosen depending on purposes, which is based on covalent and non-covalent bonds, respectively (Figure 1b). Biomaterial applications attractive to cell culture scaffolds [30,33] and drug delivery systems [91,92] have been reported using peptide fiber-based hydrogels with various bioactivities. This review focuses on the current progress in cross-linked peptide fibers and their applications.

## 2. Peptide-Based Self-Assemblies

**Figure 2:**
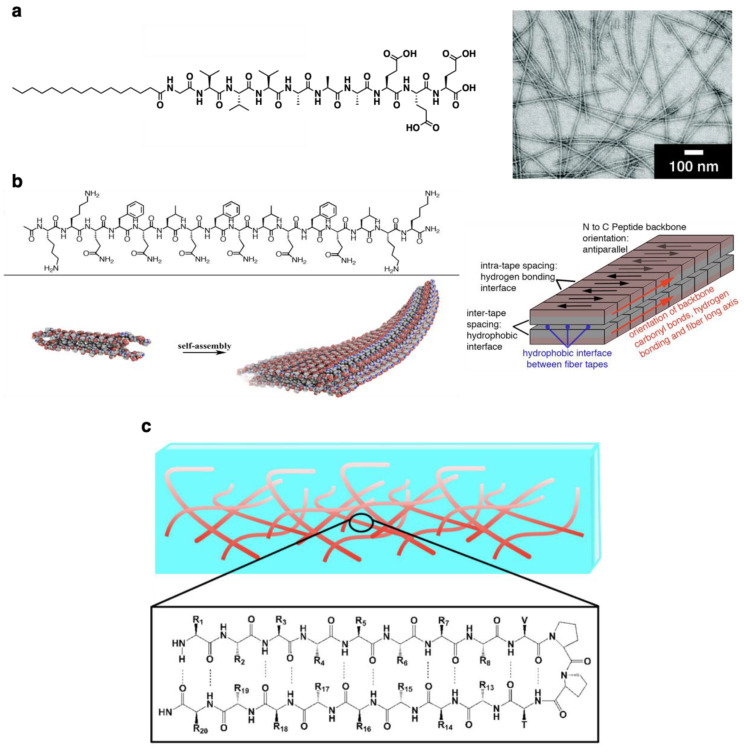
Representative self-assembled peptide-based fibers. (**a**) Peptide fibers composed of peptide amphiphiles. (**b**) Double domain-type peptide fibers containing alternate hydrophobic and hydrophilic amino acids for self-assembly. (**c**) Peptide fibers composed of *β*-hairpin peptide structures. Reprinted with permission from refs 3 (**a**), 42 (**b**), and 59 (**c**). Copyright 2008 American Chemical Society (**a**), 2013 American Chemical Society (**b**), and 2017 American Chemical Society (**c**).

Peptide-based materials can incorporate a variety of biological functions depending on the peptide sequence, and the synthetic methodology has been established on the basis of the solid-phase and solution-phase reactions. As a result of these advantages, numerous peptide-based self-assembled fibers have been developed. As a pioneer work, Stupp et al. developed peptide amphiphiles having hydrophobic chains introduced at the termini of the peptide sequence (Figure 2a). The hydrophobic part involved in the self-assembly and the hydrophilic part forming hydrogen bonds and being exposed to solutions can be designed individually, enabling the creation of peptide fibers with a variety of functions and structures. For example, kinetically stable peptide fibers are prepared when the crystalline domain is introduced to peptide fibers [1]. Recently, it was reported that the length of peptide fibers and cytotoxicity depending on the length could be controlled by introducing diene moieties at the termini of peptide fibers for polymerization after self-assembly [2]. Photofunctional peptide fibers can be designed by employing photoresponsive moieties. Photo-induced self-assemblies of peptide fibers [3] and the manipulation of cell behaviors [4] have been reported using peptides protected with a photo-cleavable nitrobenzyl group. More recently, a photo-activated mechanical actuation was realized by peptide fibers conjugated with spiropyran units in which ring-open and ring-close can be switched by a light [5,6]. As described later, a variety of manipulations for controlling cell behaviors are possible by introducing bioactive peptides into hydrophilic parts exposed to solutions, which have led to attractive applications to cell culture scaffolds and drug delivery systems. FF dipeptide, a partial sequence of amyloid fibril, is known as a minimal self-assembly unit, and various FF peptide-based self-assemblies such as fibers, tubes, and vesicles can be formed depending on conditions [40]. In particular, Ulijn reported that FF peptide having an aromatic unit, such as an Fmoc group at the terminus (Fmoc–FF peptide), forms a supramolecular fiber composed of a Fmoc–FF peptide tetramer as a repeating unit. Various functional peptide fibers can be designed by replacing the Fmoc group [34]. For example, FF peptides having an azobenzene group instead of the Fmoc group form photo-responsive peptide fibers. In addition, the introduction of anthracene or coumarin results in photo-cross-linkable peptide fibers. Enzymatic reaction-induced self-assembly of peptide fibers can be prepared by designing the molecular structure to react with enzymes. Subtilisin-responsive peptide fibers can be prepared by the methylation of carboxylic acid at the termini. Peptide fibers become phosphatase-responsive by the phosphorylation of a tyrosine part. Thermolysin and chymotrypsin-responsive peptide fibers can also be designed by coupling with condensation reactions. Peptide fibers composed of natural amino acids with high biocompatibility have recently been developed in view of practical applications. Typical examples are peptide fibers developed by Hartgerink that contain repeated hydrophilic serine (S) and hydrophobic isoleucine (L) peptide sequences forming *β*-sheets as a self-assembly unit (Figure 2b) [41,42,43,44,45,46,47,48,49,50,51,52,53,54]. Functional peptides can be incorporated to the termini of the peptide fibers forming “double domain-type peptide fibers” where the self-assembly unit and the functional part can be designed individually. Although the SL repeat sequence is commonly used as a self-assembling unit, double domain peptide fibers in which some serine residues are replaced with glutamine or dopamine have also been reported [41,42]. It was also confirmed that peptide fibers were formed, even when leucine is replaced to phenylalanine, tyrosine, or tryptophan [42]. Since the self-assembly unit is composed of neutral amino acid residues, charged amino acids can be incorporated to the self-assembly part [43,44,45,46,47,48,49,50,51,52]. Peptide fibers containing repeated cationic arginine, hydrophobic alanine, anionic aspartic acid, and hydrophobic alanine residues (RADA sequence) have been reported by Zhang [55]. The combination of ionic electrostatic interactions and hydrophobic interactions enables forming peptide fibers with structural stability. As a result, cross-linked RADA-based peptide fibers form hydrogels with high mechanical strength. The mechanical strength of hydrogels can be further enhanced by replacing an alanine residue to glycine [56]. Self-assembled peptide fibers composed of *β*-hairpin structures with multiple hydrogen bonds by the introduction of a bend structure derived from repeated proline residues were reported by Schneider (Figure 2c) [58,59,60,61,62,63,64,65,66,67]. These peptide fibers exhibit unique functions differently from the other above-mentioned peptide fibers derived from the characteristic secondary structures. For instance, the peptide fibers can flexibly bend while maintaining rigidity thanks to reversible multiple hydrogen bonds. It has also been reported that the *β*-hairpin structure can capture specific metal ions such as arsenic (As) [58].

## 3. Effects of Cross-Linking

**Figure 3:**
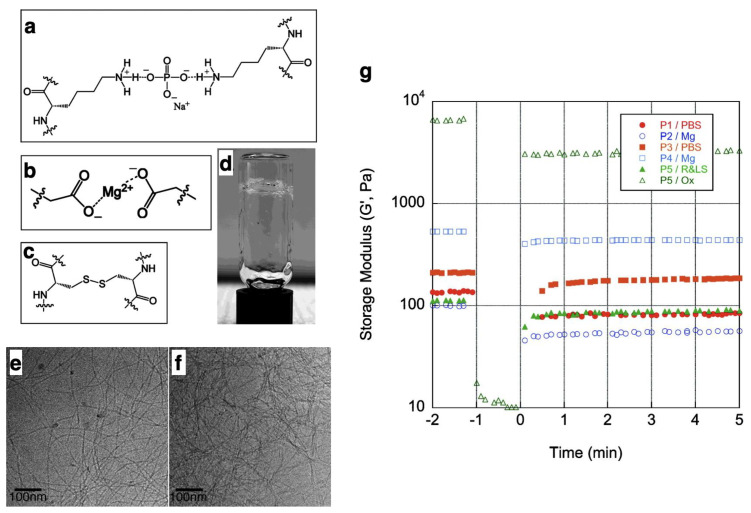
Three modes of cross-linking of peptide fibers: (**a**) phosphate cross-linking between lysine amines, (**b**) Mg^2+^ cross-linking between carboxylates of glutamate, (**c**) covalent cross-link, using cysteine, enabling the formation of disulfide bonds, (**d**) hydrogel formation by cross-linking. Cryogenic transmission electron microscopy (cryo-TEM) images of peptide fiber before (**e**) and after (**f**) cross-linking by disulfide bonds. (**g**) Shear recovery of storage modulus of cross-linked peptide hydrogels. Shear begins at t = −1 and is released at t = 0. Peptide hydrogels recovered considerable within 1 min (t = 1). Reprinted with permission from ref 53. Copyright 2009 American Chemical Society.

The cross-linking of water-soluble polymers forming three-dimensional networks can lead to changes in the physical and chemical properties of the materials. A typical change is hydrogelation, which enables the design of attractive biomaterials, including cell culture scaffolds. Conventionally, the effects of cross-linking have been investigated using covalent polymers [68,69,70,71,72,73,74,75,76,77,78]. Mechanically robust nanocomposite hydrogels can be prepared by the use of clay nanosheets at cross-linking points [68,69]. Properties of nanocomposite hydrogels can be tuned by designing interactions between clay and polymer networks. For example, nanocomposite hydrogels with high water content and mechanical strength were formed by employing interactions between dendritic macromolecules having multiple guanidinium ions and clay nanosheets [69]. Hydrogels with mechanical anisotropy were also formed by replacing clay nanosheets with magnetically alignable titanate nanosheets [70,71,72]. Double network hydrogels embedding two different types of polymer networks give hydrogels with enhanced mechanical strengths [73,74]. When the double network hydrogels are damaged, the first polymer network absorbs the impact energy and prevents breakage of the second polymer network. Slide-ring hydrogels composed of polyethylene glycol (PEG)-based networks where two connected cyclodextrins are utilized as cross-linking points are known to exhibit extremely high stretchability [75]. Since PEG can move though the cavity of cyclodextrins, cross-linking points of PEG–cyclodextrin complexes rearrange to soften the impact force on cross-linking points, preventing fracture of the hydrogels. Stimuli-responsive hydrogels can be prepared by designing association/dissociation events at cross-linking points. The above-mentioned cyclodextrin in slide-ring hydrogels forms an inclusion complex with various hydrophobic molecules. For instance, sol–gel transitions are induced by light in hydrogels embedding complexes between cyclodextrin and azobenzene as cross-linking points, where the photo-isomerization of azobenzene units causes a dissociation from cyclodextrin [76]. When inclusion complexes between cyclodextrin and ferrocene are adopted as cross-linking points, sol–gel transitions are induced in response to redox reactions by taking advantage of reversible association/dissociation events of the ferrocene moieties [77]. Hydrogels incorporating antibodies at cross-linking points have been reported in response to targets specifically. When antigens are infiltrated into the hydrogels, cross-linking points are formed by antigen–antibody interactions leading to a contraction of the hydrogels [78]. Since antibodies can be chosen as target antigens, various target-recognizing hydrogels can be designed depending on the purpose. In addition, target-sensing systems can be incorporated by the introduction of colloid crystals exhibiting structural colors, as the structural colors change in response to changes in distance between colloidal particles when the contraction of hydrogels is induced in the presence of antigens. As cross-linking is also useful in designing functional materials composed of non-covalently assembled peptide fibers, various cross-linked peptide fibers have been developed. Disulfide bonds in cysteine residues and electrostatic interactions with lysine or aspartic acid residues have frequently been utilized as cross-linking points [53]. As can be seen in Figure 3, hydrogels are formed after the cross-linking of peptide fibers, and the storage modulus (G’) of the chemical hydrogel is higher than the physical gels (Figure 3g). In addition, more sophisticated cross-linking methods have recently emerged using non-natural amino acids. In general, cross-linking methods are classified into two categories: chemical cross-linking by covalent bonds and physical cross-linking by non-covalent bonds. Chemical cross-linking by strong covalent bonds is advantageous to form hydrogels with high mechanical strength, while physical cross-linking by reversible non-covalent bonds can provide unique functions such as self-healing and stimuli responsiveness.

## 4. Cross-Linked Self-Assembling Peptides

### 4.1. Covalent Approach

**Figure 4:**
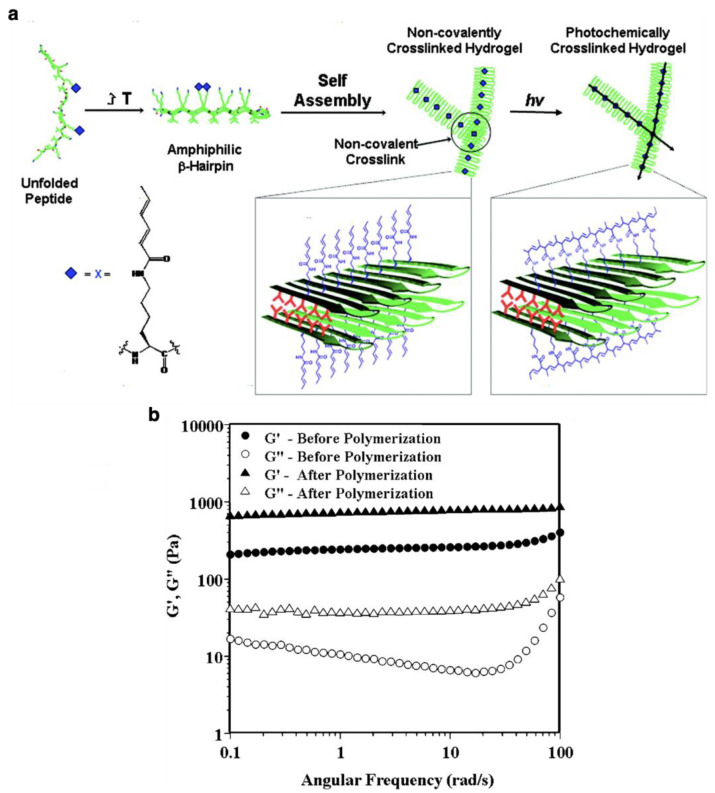
(**a**) Proposed mechanism of folding of photopolymerizable *β*-hairpin peptide fibers and their self-assembly leading to hydrogelation and the subsequent photopolymerization of its fibrillar network. (**b**) Rheological properties of the *β*-hairpin peptide fiber hydrogels before and after polymerization. Reprinted with permission from ref 67. Copyright 2010 American Chemical Society.

Covalent chemical cross-linking is an important strategy for designing biomaterials because covalently connected peptide fiber networks exhibit high mechanical strengths. In general, it is difficult to make hydrogels with high mechanical strength using peptide-based supramolecular fibers due to reversible non-covalent bonds, as compared with hydrogels composed of covalent polymers. In this sense, covalent chemical cross-linking is an effective strategy to design peptide fiber-based hydrogels with increased mechanical strength. For cross-linking of peptide fibers, reactions of amino acid side chains can be utilized by introducing thiol or catechol groups capable of forming covalent bonds by oxidation [41,53]. In particular, thiol groups allow the reversible control of cross-linking, in spite of covalent bonds, as disulfide bonds are formed by oxidation and are cleavable under reductive conditions. For example, disulfide-based cross-linked peptide fibers can be used for drug delivery, which can release encapsulated drug molecules in reductive intracellular environments. As an example of cross-linked peptide fibers using catechol moieties, double domain-type peptide fibers containing SL repeats as self-assembly units have been reported [41]. For cross-linking, some of the phenol groups of Tyr were replaced by catechol groups in the peptide fibers, and it was confirmed that the peptide fibers were stabilized by oxidation-induced cross-linking. LOX is an enzyme encoded in human LOX gene, which is activated by the cross-linked extracellular matrix by converting the amino group of lysine to highly reactive aldehydes. The cross-linking reaction is involved in the suppression of cancer metastasis and prevention of malignant transformation *in vivo*. This enzymatic reaction can be used to cross-link peptide fibers covalently by generating aldehyde groups in peptide fibers to form enamine under mild physiological conditions [54]. Transglutaminases (TGase) are enzymes that catalyze the formation of an isopeptide bond between carboxamide groups of glutamine residue side chains and amino groups of lysine residue side chains with the releasing of ammonia. It was found that TGase could also be used for enzymatic cross-linking [20]. Photo-induced cross-linking, on the other hand, allows the spatiotemporal control of the functions of peptide fibers for a variety of applications. Coumarin, an aromatic lactone derivate, undergoes a reversible dimerization reaction upon photoirradiation, and peptide fibers can be photochemically cross-linked by the introduction of coumarin units. Indeed, it was reported that peptide fibers composed of lysine dipeptide possessing two coumarin molecules are stabilized by photo-cross-linking [35]. Conjugated dienes polymerize upon photoirradiation, and photo-cross-linkable fibers can be formed using diene-tethered peptides. It was reported that covalently polymerized peptide fibers are formed by the photoirradiation of diene moieties, which were introduced to the peptide chain using computational science (Figure 4a) [67]. After polymerization, the storage modulus of peptide hydrogel was 10-fold higher than that of hydrogel before polymerization (Figure 4b).

### 4.2. Non-Covalent Approach

**Figure 5:**
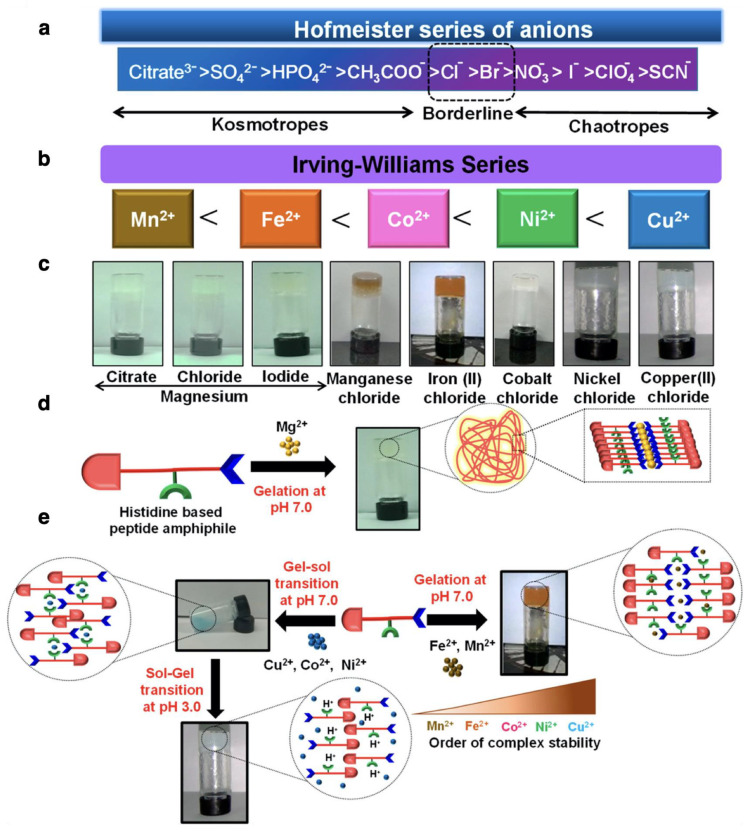
Schematic representation showing (**a**) Hofmeister series of anions, (**b**) Irving–Williams series depicting the order of stability of metal–ligand complexes. (**c**) Optical images of the peptide hydrogels of Fmoc–HL–COOH formed in the presence of metal salts at different pH. Scheme representing the variation in self-assembling behavior of histidine-based peptide amphiphile in the presence of (**d**) magnesium salts and (**e**) transition metal salts. Reprinted with permission from ref 38. Copyright 2019 American Chemical Society.

Non-covalent physical cross-linking allows us to design a variety of peptide fiber networks thanks to the dynamic supramolecular binding modes and reversibility. Electrostatic interactions have frequently been introduced at cross-linking points. In particular, the cationic amino group in lysine residue can form a cross-linking point with polyvalent anions. Cross-linked peptide fibers prepared using phosphate ions in buffer solutions or water-soluble anionic drug molecules at cross-linking points have been reported [53]. Cross-linking methods by coordination bonds between metal ions and amino acid side chains have been actively investigated due to the diversity of binding modes. In the case of natural amino acids, histidine, cysteine, and glutamic acid residues can be utilized for metal coordination [79,80,81,82,83,84,85,86]. The imidazole moiety in histidine residue is known to bind with Zn^2+^, Cd^2+^, Cu^2+^, Ni^2+^, and Ag^+^ ions [79]. When peptides include the HH sequence, the polymerization of peptides is induced in the presence of Zn^2+^ ions to form amyloid-like fibers. Especially, by introducing HH sequence to collagen peptides, a variety of microstructures were formed by hierarchical peptide assembly depending on metal ions [80]. In addition, the gelation behaviors of HL dipeptide modified with an Fmoc group at the N-terminus (Fmoc–HL–COOH) upon the addition of divalent metal ions were investigated [38]. In this case, homogeneously cross-linked hydrogels were formed in the presence of Fe^2+^ and Mn^2+^ ions rather than Cu^2+^ and Ni^2+^ ions, although Fe^2+^ and Mn^2+^ ions are known to weakly bind with the ligand according to the Irving–Williams series (Figure 5). According to the hard and soft acids and bases (HSAB) theory, the thiol group in cysteine residue and the carboxyl group in glutamic acid residue are categorized as a soft base and a hard base, respectively. Therefore, the thiol group strongly binds to the soft acids of Hg^2+^, Pb^2+^, Zn^2+^, and Cd^2+^ ions, while the carboxyl group preferentially binds to the hard acids of La^3+^, Yb^3+^, and Ca^2+^. The cross-linking methods of the *α*-helix coiled-coils using metal coordination have been reported [84,85,86]. More diverse metal coordination is available by introducing non-natural amino acids. Pyridine rings work as organic ligands to form strong coordination bonds with metal ions. To date, it was revealed that peptides can form coordination bonds with metal ions such as Fe^2+^, Re^+^, Tc^+^, Cu^2+^, Pd^2+^, Ag^+^, and Pt^2+^ by incorporating amino acids conjugated with pyridine, bipyridine, or terpyridine moieties [87,88,89,90]. A specific metal coordination can be achieved via the secondary structure of peptides. For instance, *β*-hairpin peptides show strong binding to As^3+^ ions due to the folded conformations [58]. Cross-linking methods based on hydrophobic interactions have also been used. *β*-Hairpin peptide fibers whose hydrophobic amino acid residues were used for cross-linking and peptides containing polymers with hydrophobic domains for cross-linking have been reported [59,93,94]. Molecular recognitions by hydrophobic moieties in *β*-sheet peptide fibers were also used for cross-linking. In this case, it was revealed that the volume of the hydrophobic part and existence of aromatic groups played important roles in forming rigid peptide fiber hydrogels [95].

## 5. Applications

### 5.1. Cell Scaffold Materials

**Figure 6:**
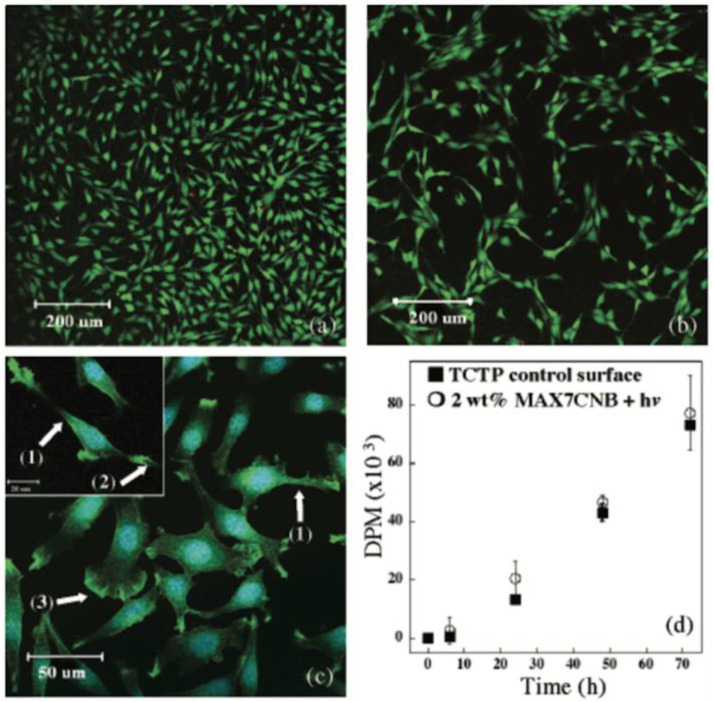
(**a**) Live/dead assay, (**b**) identical assay, (**c**) fluorescent imaging, and (**d**) rates of cell proliferation measured by [^3^H]thymidine uptake of NIH3T3 fibroblasts seeded onto *β*-hairpin peptide fiber hydrogels. In (**c**), (1) Lamellipodia, (2) filopodia of cells, and (3) ruffled membrane of a migrating cell are observed. Reprinted with permission from ref 64. Copyright 2005 American Chemical Society.

The hydrogels of cross-linked peptide fibers are materials with high water content close to physiological conditions with self-standing and moldable properties. Together with the capability to incorporate a variety of bioactive peptide sequences, cross-linked peptide fibers can provide promising platforms for cell culture scaffold (Figure 6). In order to facilitate cell adhesion to peptide fiber networks, the RGD epitope sequence is often introduced to the peptide fibers. RGD is a partial sequence of cell-adhesive fibronectin protein, and it can specifically interact with a cell surface receptor integrin. In many cases, peptide fibers become cell adhesive if an RGD sequence is introduced to the peptide sequence exposed to aqueous media. For example, the cell adhesive peptide amphiphile [21], Fmoc–FF-based peptide fibers [39], peptide fibers with RADA repeat [57], and peptide fibers with *β*-heparin structure [66] have been prepared by the introduction of an RGD sequence. However, the position of introducing the RGD sequence can affect cell adhesivity. When the RGD sequence is introduced to peptide fibers having a RADARADARADARADA sequence as a self-assembly unit by replacing RAD with RGD, peptide fibers containing the RGDARADARADARADA sequence show weaker cell adhesivity compared to peptide fibers containing the RADARADARADARGDA sequence [57]. As the rigidity of peptide fibers and rearrangement of monomer peptide units influence the viability and differentiation of cells cultured on the peptide fibers, the dynamic nature of peptide fibers has been investigated in detail. Rigidity inside peptide fibers was evaluated by spin-labeling of the molecular skeleton of amphiphilic peptide fibers with a nitroso radical [8]. In addition, super-resolution microscope revealed that monomer units of peptides in the peptide amphiphile are rearranged by exchange between fibers [19]. Peptide fibers enabling the manipulation of cell behaviors can be developed by the introduction of bioactive peptide sequences. The IKVAV sequence is a partial peptide sequence of laminin, which is a major component of the basement membrane. The IKVAV sequence facilitates cell adhesivity, cell spreading, cell proliferation, collagenase IV synthesis, and even the differentiation of nerve cells. It was revealed that the differentiation of neuronal cells could be facilitated upon the addition of peptide fibers containing the IKVAV sequence [14]. It was also confirmed that differentiation was efficiently induced by the accumulation of IKVAV units around receptors on the cell surface taking advantage of the rearrangements of monomeric peptides in the supramolecular fibers. Transforming growth factor-*β*1 (TGF-*β*1), which is produced in cells in many tissues including kidney, bone marrow, and platelets, can promote the proliferation of osteoblasts and synthesis and the growth of connective tissues such as collagen, while it suppresses the growth of epithelial cells and osteoclasts. It was reported that peptide fibers with the TGF-*β*1 binding HSNGLP sequence facilitated cartilage regeneration [16]. Vascular endothelial growth factor (VEGF) is a series of glycoproteins involved in angiopoiesis and vascularization. VEGF is a ligand that binds to vascular endothelial growth factor receptor (VEGFR) on the surface of vascular endothelial cells, which stimulates cell division, migration, and differentiation, and enhances the vascular permeability of small blood vessels. VEGF is also related to the activation of macrophages, vascularization in a normal body, and malignant processes of cancer such as tumor angiogenesis and metastasis. By designing peptide fibers containing the VEGF-mimic KLTWQELYQLKYKGI sequence, peptide fiber-based hydrogels capable of regenerating ischemic tissue can be prepared [23]. Angiogenesis is a biological event in which new branches of blood vessels construct vascular networks. It occurs in a process of wound healing and plays important roles in chronic inflammation and the growth of malignant tumors. Revascularization therapies for the relaxation of ischemia would be realized by controlling angiogenesis. Indeed, peptide fibers with a lysine repeat sequence can induce angiogenesis in mice [43]. Tissue factor (TF) is a single-chain glycoprotein bound to the cell membrane, and it works as an initiation factor for extrinsic blood coagulation reactions. TF is expressed in the outer membrane of the vascular wall of extravascular tissue when vascular injury causes blood loss. TF forms a complex with factor VII or factor VIIa to activate factors IX and X, which leads a cascade reaction to activate coagulation protease. As a result, thrombin is generated from prothrombin, which leads to the formation of fibrin gel and activates platelets at the site of vascular injury to form a blood clot. The blood coagulation reaction is closely related to wound healing, and the expression of TF is accelerated in monocytes accumulating inflammation in vascular injury and stimulated endothelial cells. In order to control these TF-related biological events, peptide fibers with TF-binding EGR or RLM sequences were designed [22]. It was confirmed that blood loss in liver tissue was suppressed by adding these TF-binding peptide fibers. Peptide fibers displaying bioactive saccharides have also been reported [11,15]. Glycosaminoglycans (GAGs) are heterogeneous polysaccharides ubiquitously found in mammalian tissues. GAG can bind a variety of proteins such as galectin, bone morphogenetic protein (BMP), fibroblast growth factor (FGF), VEGF, Sonic Hedgehog (Shh) protein, and Noggin protein to control many biological events. It has been revealed that peptide fibers displaying *N*-acetylglucosamine (GlcNAc) moiety could bind to galectin-1, enabling the modulation of bioactivities of galectin-1 [11]. It was also found that the chondrogenic differentiation of mesenchymal stem cells could be enhanced by designing glucose-containing peptide fibers [15]. Orientations and crystallizations are also important elements to design functional cell scaffold materials. Thanks to the dynamic rearrangement of molecules, even highly ordered structures can be constructed by supramolecular peptide fibers [27,28,29]. In the presence of hyaluronic acid, microscopically anisotropic peptide fiber-based hydrogels are formed through orientations of the fibers in the self-assembly process [28]. Anisotropic tissues embedding oriented cells can be prepared by culturing the cells in the anisotropic peptide fiber hydrogels. For instance, anisotropic neuronal tissue embedded in the hydrogels transmits nerve signals in one direction. Calcium phosphates, such as hydroxyapatite, are a main component of bone, and they are also used as a major component of artificial bone, as they have the capability to facilitate bone formation and osteoconductivity. Peptide fibers that can control the crystallization of calcium phosphate are applicable to regenerative medicine for bone tissue. It has been reported that crystallization is induced by peptide fibers with phosphate groups in the peptide chain to cross-link them with calcium ions [17]. Practical applications of the peptide fibers have been demonstrated using peptides fully consisting of natural amino acids, such as double domain-type peptides and peptides containing a RADA repeat sequence. For example, it has been reported that human nerve cells can be cultured with peptide fiber-based hydrogels containing the LKLK repeat sequence as a self-assembly unit [37].

### 5.2. Drug Delivery System

**Figure 7:**
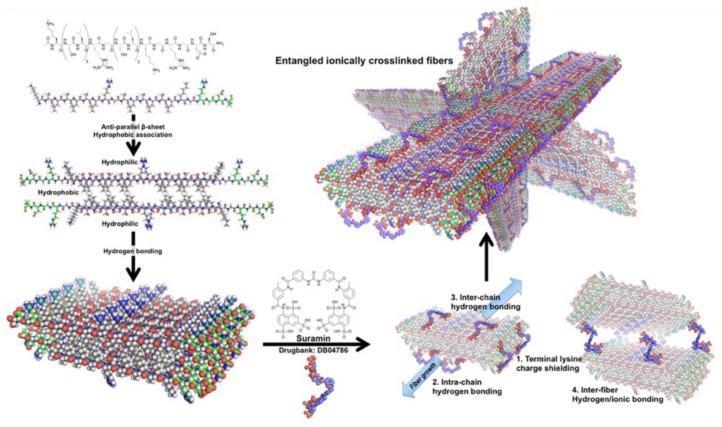
Schematic of molecular self-assembly of peptide fibers with an RGDS terminal sequence. Main SL-repeat regions arrange antiparallel to each other and hydrogen bond into sheets. Polyvalent suramin allows for intramolecular and intermolecular hydrogen bonding and ionic interaction with terminal lysines. Stabilized short fibers’ terminal charges allow fiber growth and polymerization into hydrogel networks. Reprinted with permission from Reference 49. Copyright 2015 American Chemical Society.

Cross-linked peptide fibers are also promising biomaterials for drug delivery systems (DDS). As some peptide sequences show pharmaceutical activity, they can be slowly released by the introduction of these sequences to peptide fibers. For example, peptide fibers containing the KLAK repeat sequence, which is a sequence toxic to cancer cells, can induce cell death by incubation with cancer cells [13]. Peptide fibers can induce cell death without using physiologically active peptides. The induction of cell death by changing the physical properties of peptide fibers was reported, because cell viability is affected by the rigidity and length of peptide fibers as mentioned above. For example, long peptide fibers prepared by cross-linking using polymerizable diene units at a terminus of hydrophobic chain of peptide amphiphiles enhance cytotoxic effects [2]. Diacetylene units can also be used for the polymerization of peptide fibers [10]. In addition to the anticancer effects, peptide fibers can be designed to have antibacterial activity. It has been reported that the *β*-hairpin peptide fibers containing the KV repeat sequence exhibited antibacterial activity [60]. The mechanical strength of *β*-hairpin peptide fiber-based hydrogels can be tuned to maintain the antibacterial activity by replacing the cationic residues of Lys to Arg. Indeed, the storage modulus of *β*-hairpin peptide fiber-based hydrogels containing RVRVRVRV sequences is more than double when compared with hydrogels containing KVKVKVKV sequences [61]. Not only peptide-based drugs, but small molecule-based drugs can be utilized for DDS. Hydrophobic drugs can be encapsulated in peptide fibers containing hydrophobic regions. For example, the double-domain peptide fibers, in which an SL repeat sequence forming a hydrophobic surface is included as the self-assembly unit, can encapsulate hydrophobic drugs such as SN-38, diflunisal, etodolac, daunorubicin, levofloxacin, and norfloxacin for DDS [47]. SN-38 is a topoisomerase I inhibitor with antitumor effects, and diflunisal is a salicylic acid derivative with analgesic and anti-inflammatory activity. Etodolac is a non-steroidal anti-inflammatory drug. Daunorubicin is a drug used in chemotherapy for acute myelogenous leukemia (AML), acute lymphocytic leukemia (ALL), chronic myelogenous leukemia (CML), and Kaposi sarcoma. Levofloxacin is a new quinolone-based synthetic antibacterial drug for various bacterial infections and is used for acute bacterial sinusitis, lungs inflammation, urinary tract infections, chronic prostatitis, and a certain type of gastroenteritis. Norfloxacin is a new quinolone-based synthetic antibacterial agent. These drug molecules are already commercially available; thus, DDS enabling the sustained release of these molecules would lead to further applications. A sustained release rate can be controlled by making a void in the hydrophobic region for the encapsulation of drug molecules. When double domain-type peptide fibers are designed with an SLSLSASASLSL sequence as the self-assembly unit, where some leucine residues in the conventional SLSLSLSLSLSL sequence are replaced with alanine, the drug molecules are stably encapsulated, allowing for a long-term sustained release [47]. Peptide fiber-based DDS using electrostatic interactions between cationic peptide fibers and charged hydrophilic drug molecules can be also designed (Figure 7) [49]. When cationic peptide fibers are cross-linked with anionic drug molecules of suramin or clodronate, the drug molecules are slowly released from cross-linked peptide hydrogels. Cross-linked peptide fiber networks allowing for the sustained release of the drug molecules may provide new opportunities for treating these diseases. Recently, cancer immunotherapy that enables the treatment of cancer by enhancing the immune response to cancer cells has emerged. Cyclic dinucleotides are a class of anionic immune-inducing agents. Hydrogels injectable to disease sites with the sustained release of cyclic dinucleotides can be prepared by cross-linking with cationic peptide fibers. In fact, hydrogels composed of double-domain peptide fibers carrying multiple cationic lysine residues cross-linked with an anionic artificial cyclic nucleotide of ML RR-S2CDA exhibit the effects of cancer immunotherapy not only in in vitro systems, but also in in vivo systems using mice [44].

## 6. Conclusions

Here, we have reviewed the recent progress in cross-linked peptide fibers. Cross-linking strategies that have been developed in the materials science of covalent polymers are also useful for non-covalent peptide-based supramolecular fibers. Although the initial molecular design of peptide fibers was simply attaching hydrophobic chain to hydrophilic *β*-sheet-forming peptide domains, various peptide-based self-assembly units have recently been discovered, enabling the design of more practical peptide fibers. In addition, cross-linking methods are not limited to the chemical reactions in natural amino acid residues, and various methods such as photo-induced cross-linking and metal coordination using non-natural amino acids are currently available. By taking advantage of biocompatibility and tunable bioactivities, peptide fibers have been used as biomaterials mainly for in vitro applications such as cell culture scaffolds at present. Based on the fundamental studies, applications of the bioactive peptide fibers will be expanded widely to not only in vitro but also in vivo and clinical directions in future.
